# Symmetry induced semimetal-semiconductor transition in doped graphene

**DOI:** 10.1038/srep19115

**Published:** 2016-01-19

**Authors:** Hansika I. Sirikumara, Erika Putz, Mohammed Al-Abboodi, Thushari Jayasekera

**Affiliations:** 1 Department of Physics, Southern Illinois University, Carbondale, IL 62901 USA; 2 Materials Technology Center, Southern Illinois University, Carbondale, IL 62901 USA

## Abstract

Substitutional chemical doping is one way of introducing an electronic bandgap in otherwise semimetallic graphene. A small change in dopant arrangement can convert graphene from a semiconducting to a semimetallic state. Based on ab initio Density Functional Theory calculations, we discuss the electron structure of BN-doped graphene with Bravais and non-Bravais lattice-type defect patterns, identifying semiconducting/semimetallic configurations. Semimetallic behavior of graphene with non-Bravais lattice-type defect patterns can be explained by a phase cancellation in the scattering amplitude. Our investigation reveals for the first time that the symmetry of defect islands and the periodicity of defect modulation limit the phase cancellation which controls the semimetal-semiconductor transition in doped graphene.

Graphene is a natural two-dimensional honeycomb lattice of sp^2^ hybridized C atoms, which can be thought of as two interpenetrating triangular sublattices, A and B^1^. Graphene shows extraordinary properties with potential applications in various fields^2^,^3^,^4^,^5^,^6^. Amidst spectacular properties, the simplest symmetric hexagonal topology results in a vanishing electronic bandgap at the Dirac point, one of the major obstacles which hinders the applications of graphene in digital electronics^7^. Unique electronic properties as well as vanishing electronic bandgap of graphene are inherited from its high-symmetric topology. Therefore, translational or sublattice symmetry breaking, which are achieved by various methods such as graphene nanoribbons, substrate-induced effects^7^,^8^, vacancies^9^,^10^, adsorption of foreign atomic species^11^, substitutional chemical doping^12^,^13^, etc. have been proposed to induce a sizable electronic bandgap of graphene. Breaking the symmetry of hexagonal lattice of graphene by coordinating with similar foreign atomic species (substitutional chemical doping) for creating an electronic bandgap is the focus of this work. Bandgap opening of graphene by various dopant types has been studied in the past^14^. Boron Nitride (BN), which is an isoelectronic to graphene and has a similar honeycomb topology, yet with a broken mirror symmetry which results in a wide bandgap semiconductor is an ideal dopant candidate for electronic band engineering in graphene^15^.

Doping graphene with B or N and codoping with BN have shown to be experimentally feasible^16^,^17^,^18^. Most of the experiments suggest that a phase separation of hexagonal BN (*h*-BN) islands is favored over atomically separated B and N defects; i.e. *h*-BN islands are more stable in graphene than isolated B and N scatterers, while the latter is also possible to occur^16^. Experimental and theoretical efforts continue to advance to precisely control not only the B and N concentration, but also the dopant arrangement in the host lattice of graphene^19^,^20^. Controlled doping of graphene with BN is extremely important as the electronic properties of doped systems depend on the detailed atomic arrangement, a fact that has been suggested by both experiment and theory^21^,^22^,^23^.

Both *h*-BN islands as well as isolated B and N impurities convert semimetallic graphene to a semiconductor^15^,^16^,^17^. Interestingly, semimetallic behavior is also possible in the presence of B and/or N impurities, both in the form of isolated scatterers as well as *h*-BN islands^24^. In fact, a small difference in atomic arrangement changes defected graphene from a semimetal to a semiconductor, a drastic difference in the electronic band structure of these systems.

A little has been done to understand the role of symmetry and the periodicity of defect modulation on semimetal-semiconductor transition of defected graphene^10^,^15^,^24^,^25^,^26^,^27^,^28^,^29^. S. Casolo *et al.* demonstrated that substitutional defects arranged in D_6*h*_ symmetry preserve the semimetallic behavior of graphene^28^. Y.-C. Zhou *et al.* reported a “3N rule”; the impurity atoms distributed in a way that there are 3N hexagons in between the impurity sites result in a vanishing or negligible bandgap^25^,^26^. P. Rani *et al.* showed that the electronic bandgap is maximized when dopants take positions of the same sublattice and is minimized (or even closed) otherwise^29^. These studies show the possibility of breaking and restoring the symmetry of graphene lattice upon substitutional chemical doping. However, a fundamental understanding of the role of symmetry on electronic bands of defected graphene is not yet established.

In addition to the symmetry of relative defect sites, symmetry of individual defect sites also plays an important role on electronic bands of graphene. For instance, it has been shown that electronic bandgap of graphene antidot lattices depends on the shape and size of the holes^9^. In their recent study, M. Dvorak *et al.* demonstrated the sensitivity of electronic band structure of patterned graphene to the geometry of the defect modulation as well as the defect structure^30^. No studies have been focused on undertsanding the role of the shape and size of *h*-BN islands on the electronic band structure of graphene, which is discussed in this article.

## Calculation Details

We considered two types of B/N-based defects: atomically separated B and N (isolated scatterers) and hexagonal BN (*h*-BN) islands in zigzag-edged hexagonal supercells. The periodicity of supercells can in general be defined by two lattice vectors, 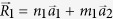 and 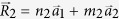. Here 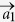 and 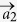 are primitive lattice vectors of hexagonal lattice of graphene. Following the notation used by Dvorak *et al.*, a supercell is denoted by four integers 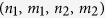
24,27. Zigzag-edged hexagonal supercells considered in this work are described by a single integer *n*, such that supercell geometry is described by the combination 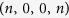. We denote the 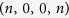 supercell by *n* × *n*. Both Bravais lattice-type patterns, where supercell lattice points are replaced by single defect sites and non-Bravais lattice-type patterns, where supercell lattice points are replaced by more than one defect site, are considered in this work.

For completeness of this work, we report the results on electronic bands of Bravais lattice-type BN-based defect patterns in graphene, which are in agreement with those from previous studies^25^,^26^. We then discuss more interesting consequences of the periodic modulation of non-Bravais lattice-type defect patterns, that lead to semimetal-semiconductor transition. More importantly our investigation reveals for the first time that the symmetry of defect islands and the periodicity of defect modulation play an important role on the semimetal-semiconductor transition in defected graphene.

All calculations were done using ab initio Density Functional Theory (DFT) as it is implemented in the *Quantum Espresso* package^31^. Zigzag-edged *n* × *n* hexagonal supercells for various values of *n* were considered. At least 10 Å vacuum space was used to separate the structure from its periodic image. Local density approximation (LDA) was used for the exchange and correlation functional with a 45 Ry energy cutoff for plane wave expansion. A 6 × 6 × 1 Monkhorst-Pack grid was used to sample the Brillouin zone. All geometries were optimized to forces less than 0.025 eV/Å.

## Results and Discussion

### Bravais lattice-type defect patterns: *periodicity of defect modulation*

In this section, we discuss how the electronic properties of graphene depend on periodicity of dopant modulation by considering Bravais lattice-type defect patterns. We considered two defect types: isolated B scatterers and *h*-BN islands in *n* × *n* zigzag-edged hexagonal periodic supercells.

#### Isolated B scatterers

**Figure 1:**
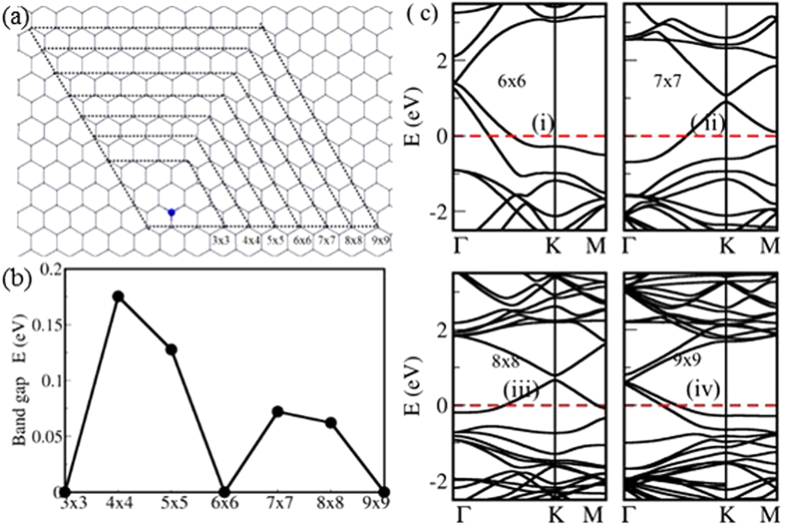
(**a**) Boron (in blue) isolated scatterers in *n* × *n* zigzag-edged hexagonal supercell configurations of graphene; *supercells of various size are marked in dashed lines*, (**b**) electronic bandgap as a function of the defect periodicity and (**c**) i–iv electronic band structures of selected boronated supercell configurations shown in (**a**). The Fermi level is marked with a red-dashed line. Dirac point falls into Γ or *K*(*K*′) point depending on the supercell periodicity.

We considered isolated B scatterers in *n* × *n* zigzag-edged hexagonal periodic supercells (for *n* = 3, to 9) as depicted in Fig. 1a. Figure 1b shows the variation of electronic bandgap at the Dirac point as a function of defect periodicity. When the defect periodicity takes the form 3*n* × 3*n* where *n* is an integer, the bandgap closes (or semimetallic behavior is observed), and the bandgap decreases when the dopant concentration is reduced otherwise^25^,^26^. Four sample electronic band structures are shown in Fig. 1c-i to iv. The Dirac point falls into either Γ point if the defect periodicity is 3*n* × 3*n* or into K (or K′) point if the defect periodicity is 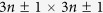
25. The bandgap opens in the latter due to intervalley scattering between two inequivalent Dirac points. When the supercell periodicity is 3*n* × 3*n*, two Dirac points merge into the Γ point destroying intervalley scattering. Thus no bandgap opening is observed. Therefore, semimetal-semiconductor transition is possible as the periodicity of defect modulation changes^24^,^25^,^26^.

#### Hexagonal BN islands

**Figure 2:**
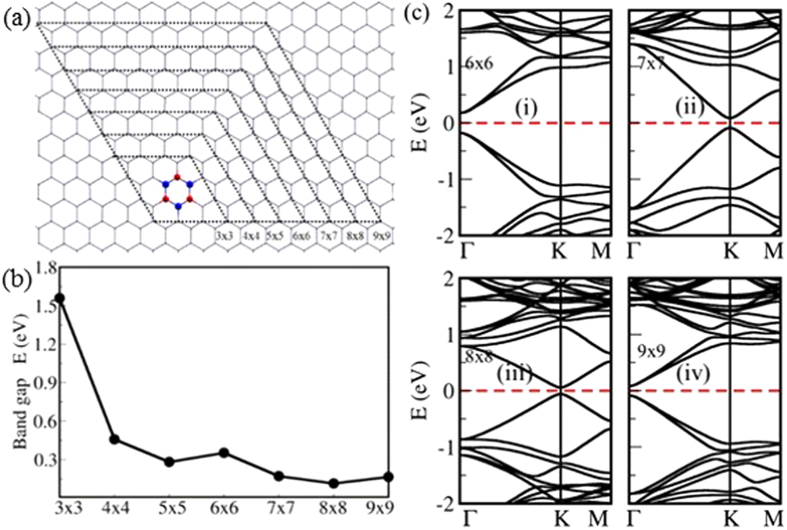
(**a**) *h*-BN islands in *n* × *n* zigzag-edged hexagonal supercell configurations of graphene – *B and N atoms are shown in blue and red respectively. Supercells of various size are marked in dashed lines*, (**b**) electronic bandgap as a function of the defect periodicity and (**c**) i–iv electronic band structures of selected configurations shown in (**a**). Dirac point falls into Γ or *K*(*K*′) point depending on the supercell periodicity.

B and N co-doping can break the A-B sublattice symmetry of host lattice of graphene; thus are attractive in graphene defects engineering^32^. We considered *h*-BN islands in zigzag-edged hexagonal supercell of graphene as shown in Fig. 2a. The electronic bandgap changes with the defect periodicity as shown in Fig. 2b, where the trend of variation is completely different from that for isolated B scatterers shown in Fig. 1b. In the previous case, with isolated B scatterers, sublattice symmetry of the host lattice of graphene is preserved. However, sublattice symmetry of host lattice of graphene is broken and no semimetal-semiconductor transition is observed in the presence of *h*-BN islands. Electronic bandgap is observed at the Γ point or *K* point depending on the supercell periodicity. The bandgap decreases with the decreasing defect concentration. However, this pattern is slightly deviated for 3*n* × 3*n* defect periodicity as shown in Fig. 2.

Within these two observations, it is clear that both translational and sublattice symmetry breaking are important in tuning the bandgap of graphene. To explore more about the effect of symmetry on bandgap tuning, we considered defects with non-Bravais lattice-type patterns^24^.

### Non-Bravais lattice-type defect patterns: *Phase cancellation of scattering amplitude*

In non-Bravais lattice-type defect patterns, more than one defect site replace the lattice points described by two vectors 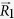 and 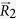. When there are more than one defect site in a supercell, we need to consider the structure factor 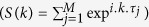 of the system for describing the electronic structure of the system^24^. Here **k** is the wave vector and *τ*_*j*_ is the position of the j^*th*^ defect site. A phase cancellation can occur for particular defect patterns, which destroys the scattering between two sublattices at the Dirac point. For a given chemical composition, a small change in the defect pattern can change graphene from semimetallic to semiconducting behavior. In order to understannd the effect of sublattice symmetry breaking and the effect of symmetry of defect sites on the semimetal-semiconductor transition, we investigated electronic structures of several non-Bravais lattice-type defect patterns in graphene.

#### Isolated B/N scatterers

**Figure 3:**
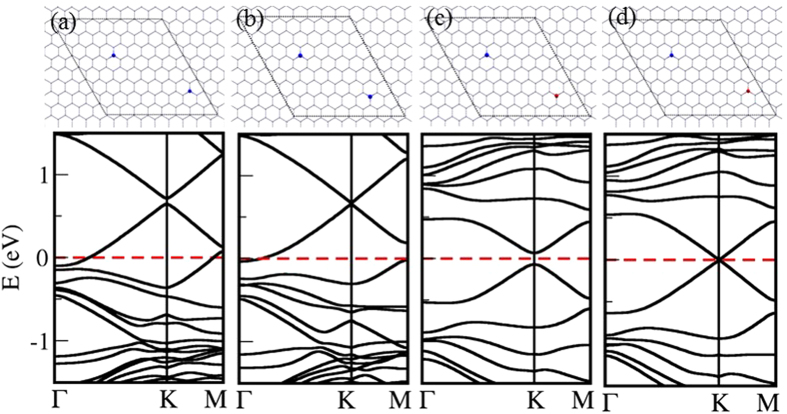
Four atomic configurations of two isolated B/N scatterers in 8 × 8 zigzag-edged hexagonal supercell of graphene with their corresponding electronic band structures: (a) two B atoms in the same sublattice, (b) two B atoms in opposite sublattices (c) B and N atoms in the same sublattice (d) B and N atoms in opposite sublattices. Dirac point falls into *K* (or *K*′) point.

We considered four cases, where there are two isolated B and/or N scatterers in 8 × 8 zigzag-edged hexagonal supercell, as shown in Fig. 3. Two C host atoms are substituted by two isolated B scatterers, which take the positions of the same sublattice in case I (Fig. 3a), and positions of the opposite sublattices in case II (Fig. 3b). In the case I, an electronic bandgap of 0.10 eV is observed at the Dirac point, and the bandgap is closed in the case II. Boron is electron deficient compared to carbon, which results in a shift in the Fermi level in both these cases. When A and B positions of the host lattice of graphene are replaced by B and N atoms respectively, 0.15 eV electronic bandgap is observed (Fig. 3c), which is closed when B and N defect atoms take the positions in the same sublattice (Fig. 3d).

When there is only a single B scatterer in the 8 × 8 zigzag-edged hexagonal supercell of graphene (Bravais lattice-type defect pattern), 0.06 eV electronic bandgap is observed (Fig. 1). Bandgap is enhanced by an additional B scatterer in the same sublattice (E_*g*_ = 0.10 eV –Fig. 3a) or an additional N scatterer in the opposite sublattice (E_*g*_ = 0.15 eV –Fig. 3c), and bandgap is closed by an additional B scatterer in the opposite sublattice (Fig. 3b) or an additional N scatterer in the same sublattice (Fig. 3d). In conclusion, a periodic pattern of single B atoms induces a scattering between two sublattices at the Dirac point opening an electronic bandgap. Sublattice symmetry broken by a single B atom can be restored by an additional B atom in the opposite sublattice or an additional N atom in the same sublattice.

#### Isolated B/N scatterers in the presence of hexagonal-BN islands

**Figure 4:**
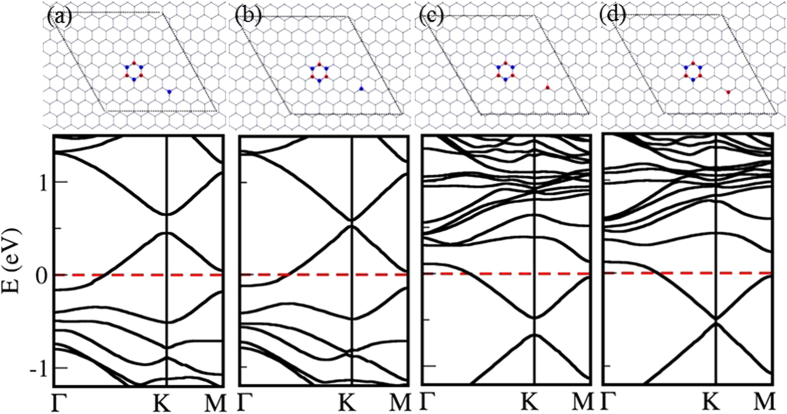
Four atomic configurations of single *h*-BN island (B and N atoms in sublattices A and B) with an isolated (a) B scatterer in the sublattice A, (b) B scatterer in the sublattice B, (c) N scatterer in sublattice A, and (d) N scatterer in sublattice B with their corresponding electronic band structures. Dirac point falls into *K* (or *K*′) point.

It is clear that breaking and restoring sublattice symmetry by selective chemical doping has a drastic effect on the electronic bandgap of defected graphene. To further clarify this point, we considered electron structure of graphene with isolated B/N scatterers in the presence of *h*-BN islands. Figure 4(a–d) show four sample configurations; all of them have one *h*-BN island in the 8 × 8 zigzag-edged hexagonal supercell. B and N atoms of the *h*-BN island take the positions of sublattice A and sublattice B respectively. In addition to the *h*-BN island, Fig. 4a configuration has an extra isolated B scatterer in the sublattice A whereas Fig. 4b configuration has an extra isolated B scatterer in the sublattice B. Even though the configurations shown in Fig. 4a,b have the same chemical composition, their electronic band structures are drastically different. An E_*g*_ ~ 0.12 eV is observed for graphene 8 × 8 zigzag-edged supercell with a single *h*-BN island (Fig. 2), which is increased with an additional B scatterer in the same sublattice (E_*g*_ = 0.20 eV – Fig. 4a), whereas an additional isolated scatterer in the opposite sublattice decreases the bandgap (E_*g*_ = 0.06 eV – Fig. 4b). This result is consistent in all of the four cases shown in Fig. 4. We further explored the idea of restoring the broken symmetry resulted by *h*-BN islands in graphene as follows.

#### More than one *h*-BN islands – *Restoring the Broken Symmetry*

**Figure 5:**
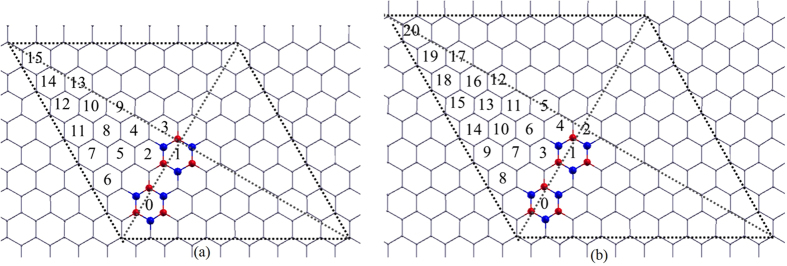
Atomic configurations for two *h*- BN islands in (a) 8 × 8 and (b) 9 × 9 zigzag-edged hexagonal supercells of graphene. One *h*-BN island takes the position marked as “0” and the second *h*-BN island takes the position marked by different numbers.

**Figure 6:**
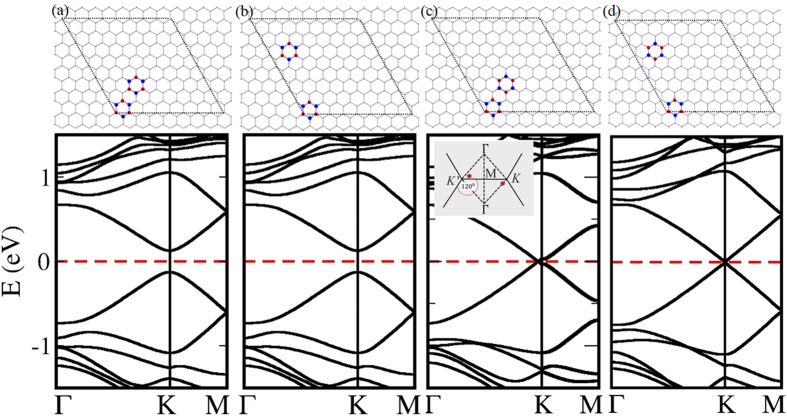
Four atomic configurations (with their electronic band structures) of two *h*-BN islands in 8 × 8 graphene supercell: B and N atoms in both islands take the same sublattice positions in (a) and (b) configurations, whereas B and N atoms of the second *h*-BN island take opposite sublattice positions compared to the first *h*-BN island in configurations (c) and (d). Dirac point falls into *K* (or *K*′) point, except for the case shown in (**c**). For the configuration shown in (**c**), Dirac point is shifted from the *K* point and falls on the Γ → *K* path. The Dirac point is also shifted from the *K*′ point. The positions of the Dirac point are marked in red dots on the inset of the panel (**c**).

As it was shown in the previous two sections, when graphene is patterned with defects, an electronic bandgap opens. This bandgap closes when there is a similar defect pattern in the opposite sublattice. Will the bandgap be closed when there are two *h*-BN islands in a supercell, and the second defect island completely restores symmetry broken by the first ? To answer this question, we calculated electronic band structures of a series of configurations with two *h*-BN islands in 8 × 8 (Fig. 5a) and 9 × 9 (Fig. 5b) zigzag-edged hexagonal supercells. One *h*-BN island takes the position marked as “0” and the second *h*-BN island takes the position marked by different numbers as shown in Fig. 5. For each chemical configuration, two atomic arrangements, *inverted* and *non-inverted*, were considered. B and N atoms of the first *h*-BN island take sublattice positions A and B respectively. In *non-inverted* systems, B and N atoms of the second *h*-BN island take the same sublattice positions as the first one, while in *inverted* systems, B and N atoms of the second *h*-BN island takes positions of the opposite sublattice as shown in Fig. 5. Our calculations show that all *non-inverted* systems with 8 × 8 configuration has a bandgap of ~0.23 *eV*, while the bandgap is closed at the Dirac point for all *inverted* systems. This suggests that the symmetry broken by the first *h*-BN island is completely restored by the second. Four sample configurations with their electronic band structures are shown in Fig. 6. It should be noted that, the Dirac point is shifted from the *K* point and falls on the Γ → *K* path for the configuration shown in Fig. 6c. The Dirac point is also shifted from the *K*′ point. The positions of the Dirac point are marked with red dots in the onset of Fig. 6c. The movement of Dirac point in the Brillouin zone due to the anisotropy of defects is explained by M. Dvorak *et al.*, in their recent paper^30^.

**Figure 7:**
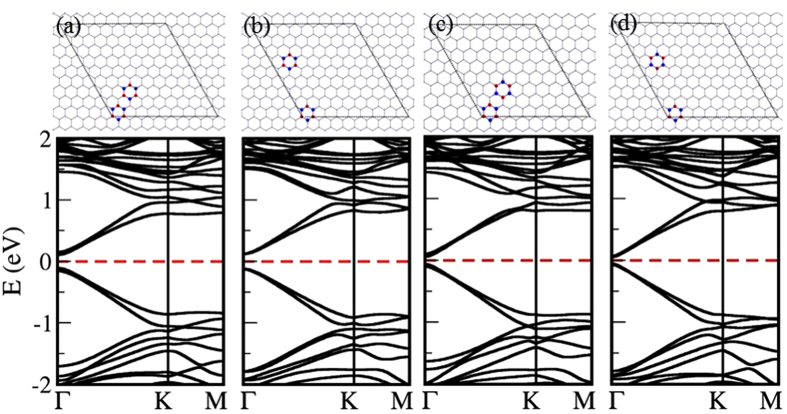
Four atomic configurations with two *h*-BN islands similar to those shown in Fig. 6, but in 9 × 9 graphene supercell. Dirac point falls into the Γ point.

The same investigation was carried out for systems with two *h*-BN islands in 9 × 9 zigzag-edged hexagonal supercell, and some sample atomic configurations with their respective electronic band structures are shown in Fig. 7. The symmetry restoring does not support to close the bandgap as it does in the case of 8 × 8 supercells. This result can be explained within the context of structure factor as it was explained by Dvorak *et al.* Phase cancellation in the structure factor, 
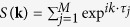
, at the Dirac point can close the electronic bandgap even if the defect periodicity suggests a bandgap opening due to sublattice symmetry breaking. In the case of 8 × 8 periodicity, the Dirac point falls in the *K* point in the reciprocal space, thus there is a possibility of having a zreo structure factor by a complete phase cancellation. However, when the Dirac point falls into the center of the Brillouin zone (where *k* = 0), it is not possible for the structure factor to be zero, irrespective of the atomic arrangement. Therefore, no semimetal-semiconductor transition is observed for *inverted* and *non-inverted* systems in the 9 × 9 systems. This suggests that a semimetal-semiconductor transition in graphene with non-Bravais lattice-type defect patterns is limited by the supercell periodicity of the system.

**Figure 8:**
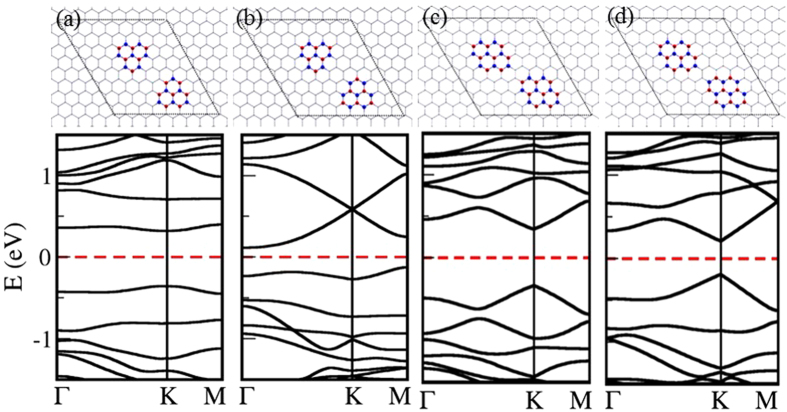
Upper panel shows atomic configurations (with their electronic band structures) of 8 × 8 zigzag-edged hexagonal supercells of graphene with two *h*-BN islands with different point group symmetries and different sublattice symmetries with their electronic band structures shown in the lower panel. Dirac point falls into *K* (or *K*′) point.

The symmetry broken by one defect site can be restored by another defect site, which results in a semimetallic defected graphene. Is it true for defects with any symmetry? Two types of defect islands in 8 × 8 periodic supercells were considered as shown in Fig. 8. The configuration shown in Fig. 8a with two *h*-BN islands both of which have C_3_ symmetry shows an electronic bandgap 0.66 *eV*. B and N atoms take positions of same sublattice in these two *h*-BN islands. Semimetallic behaviour is observed for the configuration shown in Fig. 8b, where B and N atoms take the positions of opposite sublattices. The symmetry broken by one *h*-BN island is restored by the second *h*-BN island. In contrast, the configuration in Fig. 8c with two *h*-BN islands where similar defect atoms take the same sublattice positions shows a 0.68 *eV* bandgap, whereas the bandgap does not close when similar defect atoms take positions of opposite sublattices (Fig. 8d).

A complete phase cancellation in scattering amplitude, and thus a semimetallic behavior is possible when there are more than one *h*-BN island. However, a complete phase cancellation of the scattering amplitude is limited by the symmetry of individual defect site.

### Summary

Fundamental understanding of semimetal-semiconductor transition is an outstanding problem in graphene defects engineering. Based on the results from ab initio DFT calculations, we discussed the effect of translational and sublattice symmetry breaking on semimetal-semiconductor transition in defected graphene. Electronic structure of graphene with Bravais lattice-type defect patterns has been in studied in the past. In non-Bravais lattice-type defect patterns, phase cancellation in the scattering amplitude leads to a semimetal-semiconductor transition^24^. That is, sublattice symmetry broken by one defect island can be restored by a second defect island, resulting in semimetallic defected graphene. By considering the non-Bravais lattice-type defect patterns in 8 × 8 and 9 × 9 zigzag-edged hexagonal periodic supercells, we found that the complete phase cancellation in the scattering amplitude is limited by the periodicity of defect modulation. Our result also show that, a complete phase cancellation is also controlled by the symmetry of individual defect site; individual defect cite with C_3_ symmetry supports the phase cancellation resulting in semimetallic graphene. The present results improve the fundamental understanding of semimetal-semiconductor transition of defected graphene, thus pave the path for its applications in tunable electronics.

## Additional Information

**How to cite this article**: Sirikumara, H. I. *et al.* Symmetry induced semimetal-semiconductor transition in doped graphene. *Sci. Rep.* **6**, 19115; doi: 10.1038/srep19115 (2016).
